# Fractal dimension complexity of gravitation fractals in central place theory

**DOI:** 10.1038/s41598-023-28534-y

**Published:** 2023-02-09

**Authors:** Michał Banaszak, Krzysztof Górnisiewicz, Peter Nijkamp, Waldemar Ratajczak

**Affiliations:** 1 Faculty of Physics, Adam Mickiewicz University, Poznań, Poland; 2 Faculty of Mathematics and Computer Science, Adam Mickiewicz University, Poznań, Poland; 3 Open University, Heerlen, The Netherlands; 4 Alexandru Ioan Cuza University, Iasi, Romania; 5 Faculty of Socio-Economic Geography and Spatial Management, Adam Mickiewicz University, Poznań, Poland

**Keywords:** Computational science, Applied mathematics, Population dynamics, Socioeconomic scenarios

## Abstract

Settlement centers of various types, including cities, produce basins of attraction whose shape can be regular or complexly irregular (from the point of view of geometry). This complexity depends in part on properties of the space surrounding a settlement. This paper demonstrates that by introducing a dynamic approach to space and by including an equation of motion and space resistance, a dramatic change in the stylized static CPT (Central Place Theory) image occurs. As a result of the interplay of gravitational forces, basins of attraction arise around cities, whose boundaries appear to be fractals. This study provides a wealth of spatial fractal complex images which may change the traditional understanding of CPT.

## Introduction

“Chaos always defeats order, because is better organized.” Terry Pratchett (1948–2015).

Central Place Theory (CPT) is often seen as one of the few ‘real’ theories in economic geography and regional science. Since its inception by Christaller^1^, it has prompted a wealth of publications, both theoretical and empirical (see e.g.^2–6^). CPT seeks to provide a systemic perspective on spatial order in a geographical system of settlements ranging from villages to big cities. The core idea is that the spatial distribution of market services and goods follows a rational specialisation pattern that determines the location, size and number of settlements and production places. Based on stylized assumptions on a space-economy (e.g. homogeneous spaces, even population distribution, uniform distance frictions, demand threshold levels, and competition among producers), CPT posits that a hierarchical (pyramidal) system of centers of various size classes will emerge within a hexagonal structure^7–10^. The theory has played a pivotal role in studies on spatial consumer behaviour, the retail sector, spatial product specialisation, healthcare systems, and trade and transportation analyses.

CPT has not only been applied in different domains of the space-economy, it has also developed a respectable range of different methods to test the validity of hierarchical systems in geography. Examples of such approaches are: spatial systems thinking, (hierarchical) linear programming models, rank-size rule techniques, Zipf’s law, gravitational models, distance-friction transportation models, spatial scale and agglomeration approaches, and so forth. The permanent flow of publications on CPT has brought to light the presence of quite some heterogeneity in central place systems in the real world. But how sensitive are these findings to the specific methods and models used? It is noteworthy that despite the great variety in these methods and approaches, two basic features are always present in empirical CPT tests, viz. returns to scale in urban centers and distance friction costs. But only rarely have these two basic assumptions been scrutinised in a dynamic and evolutionary geographical system. This prompts the challenging question whether it is possible to design a general theory on hierarchical spatial systems that comprises the above-mentioned static approaches as specific cases (see also^11–13^).

Despite many useful extensions of CPT, for instance, in the context of urban network theory^14^, it was mainly the static approach that dominated these works and contained significant limitations on the often stylized structure of the hierarchical network of central places. Therefore, answering the questions posed above requires incorporating nonlinear dynamics into the theory of spatial interactions. Then it is possible to establish a boundary–where the chaotic spatial interactions of the cities under study end, and orderly interactions begin–as in the case of classical CPT. Conducted in the spirit of recent works by Banaszak et al.^15,16^, this study is devoted to the dynamics of spatial interactions generated by urban units included in a regular hexagonal CPT pattern (see also: ^7–9,17–25^). It is well known from the literature that the hexagonal system is the most efficient (in terms of transport costs) space-filling configuration (see^26^). This study seeks to develop a comprehensive dynamic ‘umbrella’ methodology in which prevailing static CPT approaches can be positioned and combined from a unitary Newtonian motion perspective on hierarchical systems.

**Figure 1:**
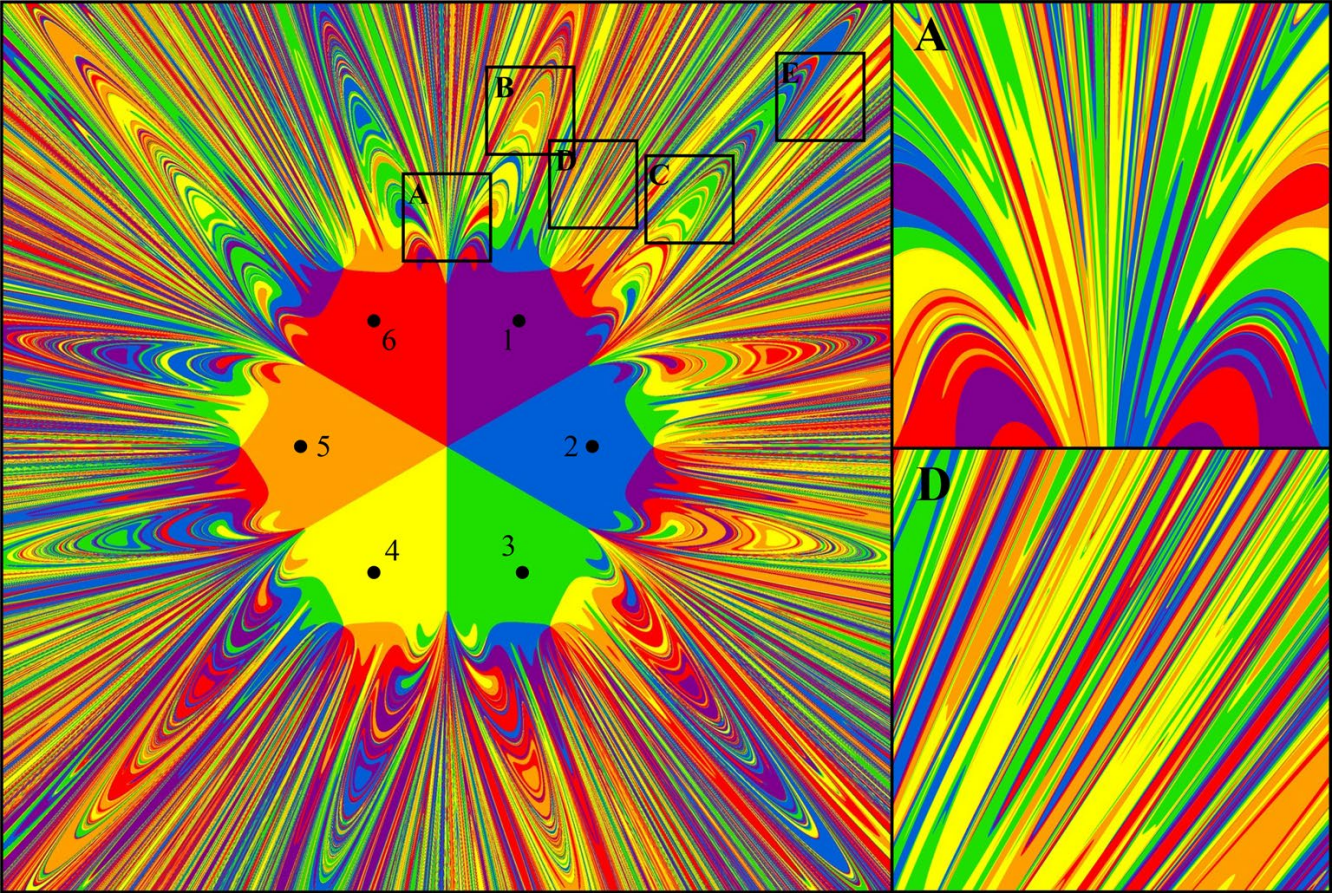
Gravitational fractals in a CPT system. Legend: Points 1–6 represent six distinct cities (with six different colors) in a hexagonal force field; (**A** and **D**) represent illustrative fragments.

Recent studies on the dynamics in hexagonal systems have shown that, in a study of spatial interactions, the transition from a static to a dynamic approach to geographical space allows one to discover irregular spatial structures which are not hexagons at all. These structures are obviously fractals. Hence, a more profound analysis requires fractal geometry methods^27,28^. These studies also revealed that, even with the simplest assumption that in the hexagonal system concerned the masses of all cities are identical, the effect of spatial interactions of these cities leads to a fractal with a very high degree of complexity and with an irregular shape, as illustratively mapped out in the archetypical Fig. 1 depicting clearly fractal dimensions in a dynamic hexagonal space based on CPT (see also Annex). This means that a static approach to the analysis of spatial interactions in the form of hexagonal lattices with overlapping force fields of cities of various sizes, as assumed in the classical theory of central places, is at least incomplete or disputable. Clearly, spatial interactions of cities regularly located on the vertices of the hexagon can be spatially represented by either a completely ordered structure or, conversely, by a completely chaotic one, with various intermediate spatial systems and different complexities^29^. The stylized assumption that all cities have the same mass is just one of many possible cases. Therefore, it can and should be tested in further experiments, both theoretically and empirically, in fractal context.

Our approach originates from the application of the Newtonian equation of motion:

$$\frac{{d}^{2}\overrightarrow{r}}{d{t}^{2}}=-\mu \frac{d\overrightarrow{r}}{dt}-\nabla U,$$

where $\overrightarrow{r}$ is the agent location vector, $t$ is time, $\mu$ is a coefficient of friction, $U$ is a gravitational potential and $\nabla$ is the gradient operator.

The attraction of an agent by one of the six cities is specified by the attributes of movement^15,30^. The agent’s trajectory is chaotic and its speed strongly depends on the distance friction $\mu$ and consequently on energy dissipation. In the model used in this study, the friction parameter μ is interpreted as a proxy for transport costs. It is worth adding that simulation experiments can also use Newtonian mechanics^31^ and Hamiltonian-Lagrange mechanics^32^.

Figure 1 is a picture of order and spatial chaos. The order represents the interior of the hexagon, with the simulation process divided into six equal sectors. They are parts of attraction basins of each of the six cities. Chaos is mapped by an array of alternating, differently colored layers representing cities attracting an agent. They have extremely complex shapes. There is always a different color layer between two different layers. This sequence of differently colored layers occurs at each zoom level and approaches infinity. This is an example of pure chaos as it is impossible to establish which city will attract the agent.

Determination of the degree of complexity of the figure depicting spatial chaos requires identification of the fractal dimension of each attraction basin boundaries which are fractals^33,34^. On the other hand, the analysis of the complexity of spatial interactions of cities in the hexagonal system of CPT differs from the static approach. Hence our study has two main goals:

1. To construct an algorithm for calculating fractal dimensions of both gravitational fractals and gravitational attraction basins with a very complex structure,
2. To analyze gravitational basins of attraction from the perspective of CPT principles.

Fractal dimensions introduced as a scientific research concept by Mandelbrot^35^ have become very popular and have generated a large number of scientific publications. They have also found several applications in spatial and urban dynamics (see e.g.,^36–^^38^). In this study, we will mainly employ model-based, visualized spatial simulation experiments in order to frame our conceptual research.

The complexity experiments performed here posit and confirm the suitability of both the *Box-counting method* as well as the *Ruler method.* The box-counting dimension is widely known and has been discussed in the literature on this subject by Falconer^39,40^, Shishikura^41^, Zatos^42^, Li, Arneodo, Nekka^43^, Fernandez-Martinez^44^, Pearse^45^, and Van den Eijnden^46^, to name a few. Its main mathematical and operational properties are summarized below in comparison to another frequently used fractal dimension, i.e., the ruler dimension.

As has been noted above, in this study the box dimension (*d*_*b*_) is used to analyze the basic complexity of gravitational fractals, and the ruler dimension (*d*_*r*_) is used for comparative purposes. In this work, the description of the theoretical foundations of these dimensions will be limited to the necessary minimum, while the algorithm for determining the Box and Ruler dimensions for structurally highly complex fractals was specifically designed by the authors.

**Figure 2:**
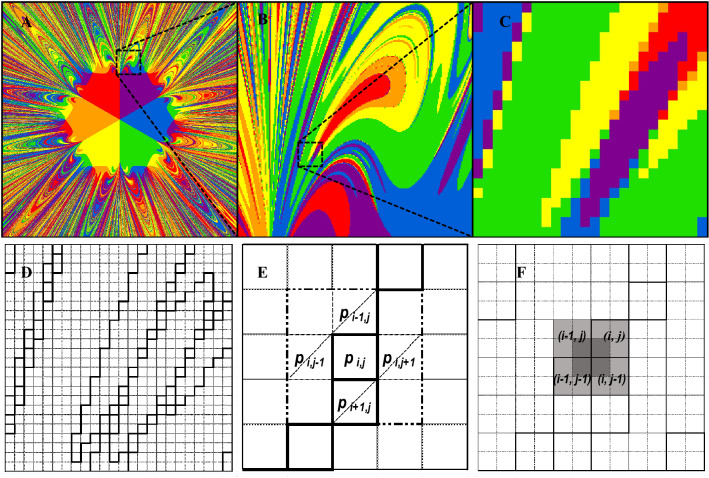
Methods of counting fractal dimensions for randomly selected magnifications of a fragment in hexagonal space. Legend: (**A–C**) Magnification of randomly selected fragment of fractal; (**D**) Boundary of attraction basins in selected fragment; (**E**) Ruler dimension determined by the 4 neighboring pixels of each boundary pixel; (**F**) Box dimension depends on 4 adjacent pixels with one of them being a boundary pixel.

Methods of determining fractal dimensions for the boundary line of cities’ gravitational basins are outlined in Fig. 2. For randomly selected fragments (Fig. 2a-c), the boundary of all attraction basins is shown in Fig. 2d (i.e. the line between figures marked with different colors).

For the box dimension (Fig. 2e), a pixel grid is created and shifted by the vector $\left[\frac{1}{2},\frac{1}{2}\right]$ with respect to the original simulation image. Then, by counting its pixels, the covering of the boundary can be determined as follows:

$${d}_{b}=\frac{\mathrm{log}\left({P}_{b}\right)}{\mathrm{log}\left(n-1\right)} ,$$

where ${P}_{b}$ is the number of all pixels that cover the boundary of the attraction basins. In the ruler dimension case (Fig. 2f), we note that direct data obtained from the simulation allow us to define the fractal dimension in the following form:

$${d}_{r}=\frac{\mathrm{log}\left({P}_{r}/2\right)}{\mathrm{log}\left(n\right)} ,$$

where ${P}_{r}$ is the number of all the sides of square pixels adjacent to the relevant boundary.

A more detailed description is provided in the Annex with Supplementary Materials, which allows the reader to fully appreciate and interpret the new conceptual framework and findings.

## Results and analyses of global and local fractal dimensions

This paper describes the complexity of gravitational fractals in terms of global and local dimensions. They are presented in Table 1.

**Table 1:** Global and local dimensions of gravitational fractals and attraction basins.

	Global cases		Local cases
	Dimension of the boundaries of all six gravity attraction basins (2.1)		Dimension of the boundary of a characteristic fragment of gravitational fractals ***A*** and ***D*** (2.4)
	Dimension of the boundary of each attraction basin from 1 to 6 (2.2)		Dimension of the boundary of each attraction basin occurring in the selected fragment of fragments ***A*** and ***D*** (2.5)
	Dimension of the attraction basin of each city as a geometric figure, which is not a fractal (2.3)		Dimension of the parts of the attraction basins treated as an irregular geometric figure, which is not a fractal (2.6)

The numbering 2.1 – 2.6 corresponds to the subdivision of the text presented in the paper.

The fractal in hexagonal CPT space, shown in Fig. 1, has a very rich structure, and therefore its characterization by means of fractal dimensions requires two approaches: (1) a global approach treating the fractal as a complex whole and (2) a local approach which allows us to determine the dimension of its fragments which are particularly interesting from a research perspective (see also Table 1). In the subsequent part of the paper, the results obtained are presented and interpreted according to the division in the table.

### Global dimension of boundaries of gravity attraction basins

**Figure 3:**
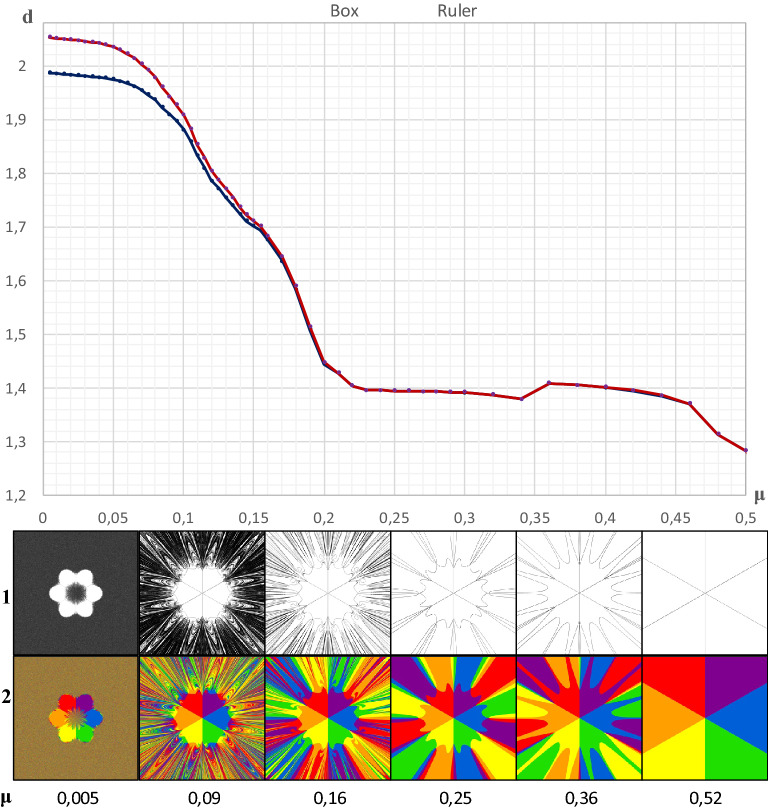
Comparison of the variability of the global ruler and box dimensions. Legend: The edge of all attraction basins is a function of the μ coefficient; 1–edges of all basins, 2–entire basins.

Two types of fractal dimensions have been thus far used in this analysis, i.e., the box and ruler dimensions. Figure 3 shows the distribution of the values of these dimensions determined for the boundaries of attraction as a function of space friction μ.

Figure 3 empirically confirms a fact known from chaos theory that whenever a fractal represents full chaos, the ruler dimension may be greater than 2 (Peitgen et al.^33^, 192–209), whereas the box dimension never exceeds this extreme value. Clearly, for a certain value of μ (in this case *μ* = 0.19), the numerical values of both types of dimensions are identical.

In the bottom part of Fig. 3, line 1 illustrates the variability of the shapes of the attraction basins of individual cities depending on the value of μ, i.e., space resistance. The initially extremely complex shapes of the boundaries are smoothed to take the form of straight lines in the case of a large value of *μ* (*μ* = 0.52).

In turn, line 2 illustrates not only the boundaries of the attraction basins, but also their internal structure. Clearly, the initially chaotic impacts of individual cities on the agent (*μ* = 0.005) are gradually smoothed out, so that in the final stage of the process they fully stabilize. This means that each city has a geometrically identical basin of attraction. Hence, if the agent is in the attraction basin of city 1 (purple color), it will always be attracted only by that city. This rule also applies to the other cities. It is obvious that the random process occurring at *μ* = 0.09 is then replaced by a strictly deterministic one. When chaos becomes complete order (Banaszak et al.^15^, the numerical values of both types of dimensions appear to stabilize at the level of 1.

### Global dimension of the boundary of each separate attraction basin

**Figure 4:**
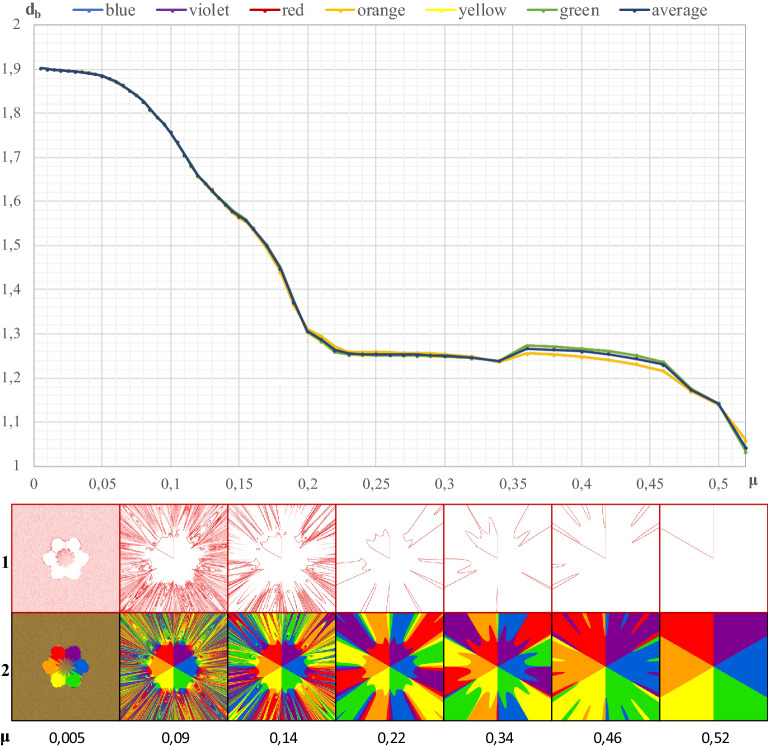
The box dimension of the edges of the attraction basins depending on the μ coefficient (separately for each attractor). Legend: 1–boundaries of single attraction basins, 2–entire basins.

Figure 1 also shows the geometric image of the attraction basins of individual cities. They were almost identical, and therefore also the fractal dimensions of the boundaries of these basins must match. The validity of this proposition is confirmed by Fig. 4. Six lines representing the distribution of the fractal dimension of the boundaries of the six basins coincide with almost full accuracy. Further analysis of Fig. 4 allows us to infer the conclusion that there is almost total chaos at the value *d*_*b*_ = 1.9021 (*μ* = 0.005). On the other hand, as space resistance increases to the value of *μ* = 0.22, there is a rapid decrease in the value of the fractal dimension of the boundary of each basin to the level of 1.2628; when μ = 0.34, then *d*_*b*_ = 1.2382. In that case, the value of the fractal dimension stabilizes, and at *μ* = 0.46, *d*_*b*_ = 1.2444 and finally for *μ* = 0.52, *d*_*b*_ reaches the value of 1.0412. The icons presented in Fig. 4 in lines 1 and 2 have slightly different structures than the icons in Fig. 3, due to different values of μ in certain cases.

### The global dimension of the attraction basin of each city as an irregular geometric figure

The full symmetry of the basins of attraction of individual cities can be disturbed by the shape of the geometric figure on which the deterministic fractal is modeled. Such a situation occurs in the present case. Due to the fact that the fractal in Fig. 1 is formed on the surface of a square, the final basins of attraction of cities 1, 3, 4 and 6 are obviously larger than those of cities 2 and 5. Of course, these differences do not occur when considering the surface inside the hexagon.

**Figure 5:**
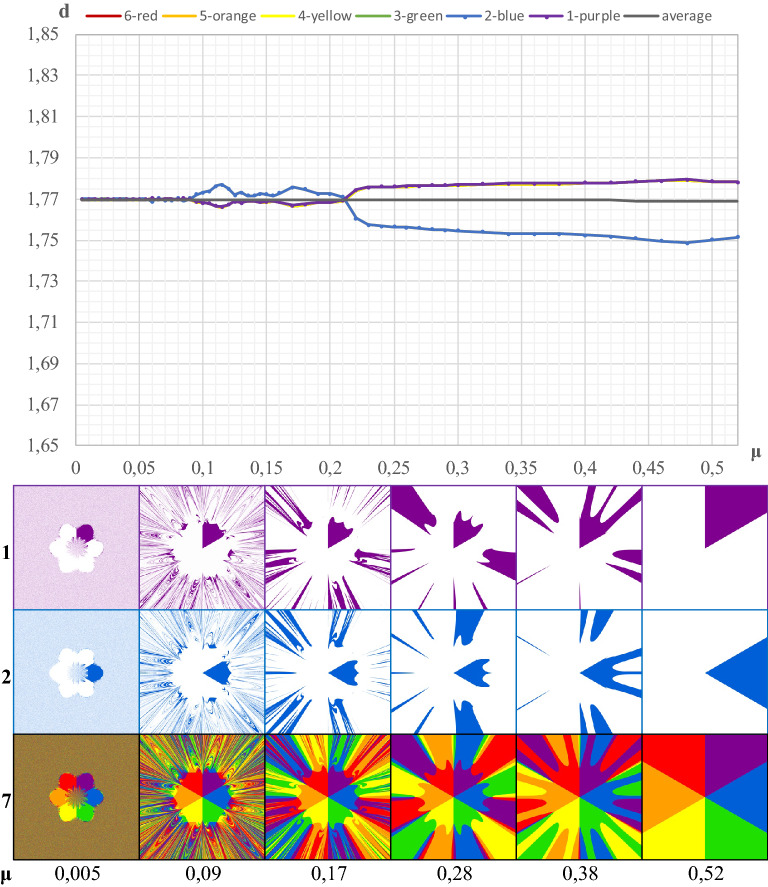
Box dimension of the attraction basins as a geometric irregular figure in the gravitational fractal. Legend: 1-basins of the first city, 2-basins of the second city, 7-basins of all cities.

In Fig. 5, the line marked in black color represents the average value of the fractal dimension of the basins of attraction of individual cities, the value of which is $\overline{{d }_{b}}=1.77$. It can be seen that at very high values of the fractal dimension in the range (1.750, 1.775), there are d_b_ oscillations around this line. This is precisely the effect of modeling the fractal on the surface of the square, rather than the properties of this fractal. Therefore, $\overline{{d }_{b}}=1.77$ should be regarded as the global dimension of the basin of attraction (of each city) treated as an irregular figure.

### Local dimensions of the boundary of the selected characteristic fragments

**Figure 6:**
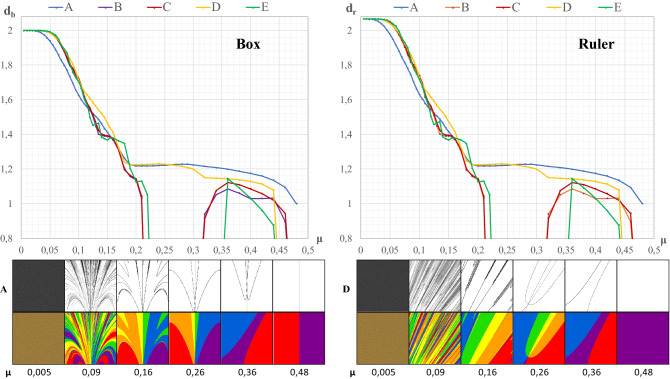
Distribution of the values of fractal dimensions of the boundaries of the attraction basins identified in selected fragments of a fractal; Legend: (***A***, ***D***)-fragments marked in Fig. 1.

Figure 6 presents fractal dimensions, with the Box and Ruler as functions of μ, and the boundaries of the attraction basins of individual cities occurring in all fragments ***A***, …, ***E***.

It is evident that the structures of Fig. 6 (Box and Ruler) are almost identical. This means that, as has been stated earlier, when describing complex fractal objects, it does not really matter which type of dimension is used.

**Figure 7:**
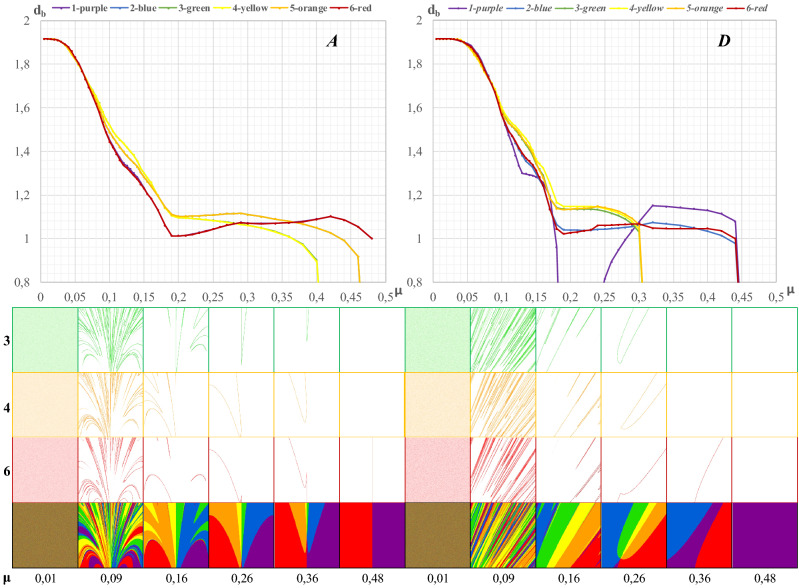
Box dimension of the edge of each gravitation basin in *A* and *D*. Legend: The icons show the variability of the fragments *A* and *D* due to the share of the attraction basins of individual cities (3, 4 and 6).

Of interest here is the variability of the structure of both figures along with the increase in the value of the parameter μ. Fragments ***A***, …, ***E*** (see Fig. 1) are characterized by high complexity, i.e. the intertwining attraction basins of the individual attractors (cities). This observation is confirmed by the numerical results of both fractal dimensions whose values are in the range (1.68–1.82). To illustrate the spatial complexity of these fragments, and thus their dimensions, by way of example, two fractal fragments are considered below: fragments ***A*** and ***D*** (see also Fig. 7).

Figure 6 offers important conclusions concerning the organization of social and economic life in the geographical area surrounding individual cities (attractors).

1. Out of all the separated fragments, only in fragment ***A*** do we find the attraction basins of all the cities intertwined across the entire range of variation *μ*, i.e. (0.00–0.48). Hence, the graph of fractal dimension (*d*_*b*_) (blue line) as a function of μ is continuous, and when the resistance of space is the greatest (*μ* = 0.48), the fractal dimension *d* = 1.00. This means that chaos has given way to total order, and fragment ***A*** has been symmetrically divided between cities 1 and 6. Hence, there are two colors left, namely red and purple.
2. A similar situation occurs in the case of fragment ***D*** (yellow line), where the attraction basins of individual cities intertwine continuously within the range: 0.00 ≤ *μ* ≤ 0.46. Beyond the value of 0.46, the entire fragment ***D*** is filled with purple: the closest city 1 dominates it.

The research conducted here also confirms the conclusions presented in previous works by Banaszak et al.^15^^,^^16^ concerning the transformation of chaos into spatial order, which means the stabilization of permanent dominance, usually of one attractor (city). Thus, with regard to fragments ***A*** and ***D***, in fragment ***A*** there is a constant dominance (in half of the area) of cities 1 and 6, from the limit value of *μ* = 0.24 onward. In the case of fragment ***D***, beginning with the value of *μ* = 0.36, only city 1 dominates (purple). That is, in the final phase of establishing the order in spatial interactions in the arrangement of areas ***A*** and ***D***, the role of the dominant attractor (city) is played by city 1 (purple).

Due to the symmetry of Fig. 1, similar effects can be observed in other parts of this fractal, located symmetrically in relation to ***A***, …, ***E*** (see Supplementary Material).

Figures 1 and 6 confirm the findings, known in the theory of city development, that urban (and other) centers rise in the hierarchy (or their rank decreases), depending on the external and internal factors conditioning their development. In the model used in this study, the parameter μ represents external factors (space resistance). If *μ* values are low, all cities are attractive from the point of view of spatial interactions and create their own but symmetrical basins of attraction. When the resistance of space increases, one city becomes the dominant center, and its basin of attraction is a uniform compact isotropic surface.

However, this is not a simple mechanism, since, as has been demonstrated by simulation experiments described in this paper, within a certain range of *μ* values, another city (attractor) may dominate the others during chaotic interactions. The dynamic history of urban development confirms this observation, for example, in relation to historical capitals of some countries that have lost their functions as administrative capitals.

### Local dimension of the boundary of each attraction basin in a selected fragment of a fractal

Fragments ***A***, …, ***E*** (Fig. 1 and the Supplementary Material) consist of mutually intertwined basins of attraction (six cities) whose boundaries with complicated courses have a fractal dimension, e.g. a box dimension.

Figure 7(fragment ***A***) shows the distribution of *d*_*b*_ as a function of μ in this fragment. In the case of total internal chaos, the fractal dimension of the boundaries of the attraction basins of all cities is identical and amounts to 1.9152. A clear differentiation of *d*_*b*_ is noticeable from *μ* = 0.1 onward. It should also be noted that orange and blue, red and purple, yellow and green lines mutually coincide. The red–purple line tend towards *d*_*b*_ = 1 as *μ* increases. However, orange, blue, yellow and green lines reach a value of *d*_*b*_ = 0.

The fractal dimension *d*_*b*_ = 1.0 is most closely represented by the blue line (city 2), then the red line (city 6) and the purple line (city 1). Since these lines almost coincide, and the red and purple lines are the last to reach the value *d*_*b*_ = 1, at *μ* = 0.48, fragment ***A*** is symmetrically covered in red and purple. Therefore, with very high spatial resistance, fragment ***A*** is dominated by two cities, namely by 1 and 6.

In turn, Fig. 7(fragment ***D***) illustrates the variability of the fractal dimension of boundaries of the attraction basins in this fragment. This dimension depends on the complexity of the mosaic patterns formed in this fragment, with varying *μ* values. When the values of *μ* are close to zero, all cities contribute to filling the space of fragment ***D***. When *μ* = 0.18, city 1 (purple color) falls out of the competition for space, but only up to the value of *μ* = 0.24, when it starts to compete again with other cities. From the point of view of spatial interactions, in the final phase of this process (*μ* = 0.44), city 2 (blue) and city 6 (red) dominate to a small extent, because cities 3, 4 and 6, starting from *μ* = 0.3, do not play any role in fragment ***D***.

Figure 7 shows that the value *μ* = 0.3 is a characteristic point. It is a locus where all the curves representing the attraction basins of individual cities meet. As has already been stated, three of them lose their influence over the space of fragment ***D***.

### Local dimensions of parts of the attraction basins treated as an irregular geometric figure

**Figure 8:**
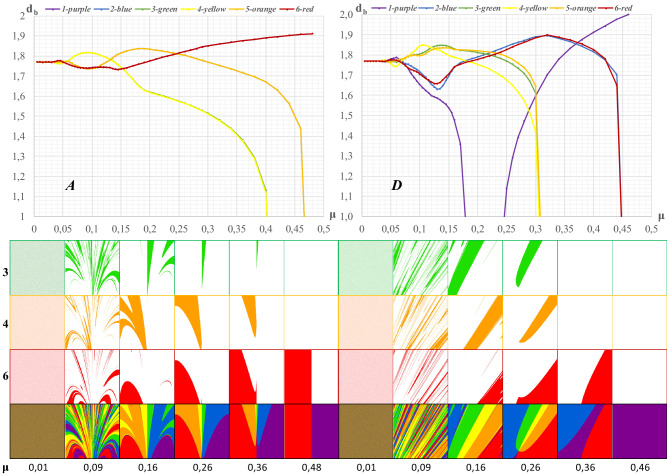
Local dimensions of parts of the attraction basins treated as an irregular geometric figure in (***A***) and (***D***). Legend: The icons illustrate the variability of the shape of some of the attraction basins of individual cities in fragment (***A***) and (***D***) for cities 3, 4 and 6.

In each of the selected fragments ***A****, …, ****E***, some of the boundaries of the attraction basins of individual cities are distributed differently. They create certain holes in the form of irregularly colored mosaic patterns that have a certain fractal dimension. To present its variability, fragments ***A*** and ***D*** were used again. Figure 8 shows the distribution of *d*_*b*_ values depending on the value of *μ*.

The function has several characteristic points. Up to the value of *μ* = 0.04, attraction basins show a jumble in which no predominant color or shape can be identified. The fractal dimension is then: *d*_*b*_ = 1.7697. From this value onwards, where *μ* = 0.042, the interior of fragment ***A*** becomes increasingly ordered. With a value of *μ* = 0.125, the city's attraction basins 3 and 4 begin to disappear in fragment ***A***. The same happens to the city attraction basins 2 and 5 for the value of *μ* = 0.24.

The final effect of the increase in space resistance (with *μ* = 0.50) leads to the filling of fragment ***A*** with two colors, i.e., purple and red. This means that cities 1 and 6, have won the competition for the space of fragment ***A***. In this case, the fractal dimensions *d*_*b*_ equal 1.90.

Figure 8 presents the variability of the fractal dimension and the effects of the competition for space between cities in fragment ***D***. As is the case in fragment ***A*** and all others, i.e. ***B***, ***C*** and ***E*** (see the Annex with Supplementary Material), the intertwined attraction basins are represented by the area consisting of an endless number of differently colored dots. Hence, up to the value of *μ* = 0.042, fragment ***D*** is dominated by pure spatial chaos that extends over its entire area. It is characterized by the fractal dimension *d*_*b*_ = 1.7697. This means that with an increase in the value of *μ*, for the emergence of an irregular shape of a geometric figure, chaos must be accompanied by an increase in the value of the fractal dimension. Its limiting value is number 2. Then, spatial dominance is usually gained by one city and the examined fragment is filled with one color (‘the winner takes it all’).

This is precisely the situation in Fig. 8 where city 1 (purple color) has apparently won the competition. Since this color fills area ***D*** completely, we find the plausible result *d*_*b*_ = 2.0.

## Conclusions

The complex spatial arrangement shown in Fig. 1 was considered in our study as a fractal from two perspectives. First, the general one represents an entire irregular geometric figure. The degree of complexity of this type of figure is defined by the fractal dimension. However, the second figure was considered from the point of view of the complexity of the boundaries of attraction basins, corresponding to individual cities (attractors). These boundaries are not simple straight lines, but within themselves they have a Cantor like a fractal structure ^47,48^.

Both approaches require establishing a precise procedure for identifying the dimensions that describe the complexity of fractals. These procedures have been presented here for the first time in the literature on the CPT principles and have been used to quantify the spatial complexity of gravitational fractals. This means that complex fractals can be comprehensively described by fractal dimensions. Thus, the analytical possibilities, concerning complex spatial systems, have been extended in the scope of our study.

Although the initial goal of the work was to construct an algorithm for calculating the fractal dimensions of complex spatial systems, the paper has a broader scope. It confirms that the spatial hierarchy of cities tends to change over time. And in the long history of the development of settlement networks, it appears that urban systems are dominated by various urban centers, but not necessarily by those which gained the dominant position in the final phase of the process^49,50^. Notably, the domination processes would always take place, even if the cities included in the hexagonal model had identical attractiveness, e.g., in terms of urban mass. Thus, the results presented here extend and even challenge the findings of the conventional CPT. In the approach adopted here, the fractal dimension is determined on the basis of 6 colors taken into account rather than 2, namely black and white.

The results presented here concerning the variability of the impact of cities on the space around them indicate the need for further work, both theoretically and empirically. In light of our findings, the following issues still need to be addressed in follow-up research:

- How has space resistance changed over time in different locations in the world and what factors have affected these changes?
- Is there an "optimal" level of space resistance from the point of view of the organization of social life in the space-economy?
- How will the range of influence of cities change as a result of global urbanization in a digital world?

Providing answers to the above questions requires intensive interdisciplinary cooperation and participation by experts in economic history, anthropology, socio-economic geography, transportation science, mathematics, econophysics, social physics and other areas of science. Fractal research may lead to the identification of a new system of laws of spatial evolution and governance of which CPT was–and maybe still is–an important element, but not an exclusive driver.

## Supplementary Information

Supplementary Information.

## Data Availability

The datasets generated during and/or analyzed during the current study are available in the Zenodo repository (.zip files). https://doi.org/10.5281/zenodo.6677995. Target URL: https://doi.org/10.5281/zenodo.6677995, https://zenodo.org/record/6677995.
