# Supplementary material for: Fractal dimension complexity of gravitation fractals in central place theory
Source: Sci Rep. 2023 Feb 9;13:2343. doi: 10.1038/s41598-023-28534-y (PMC9911407; doi:10.1038/s41598-023-28534-y)
Supplement: Supplementary file 1 — Supplementary Information. [file 41598_2023_28534_MOESM1_ESM.docx]

**Annex: Supplementary Material**

The supplementary materials include an explanation of the structure of basins of attraction of cities in a hexagonal pattern (Figure 1), the definitions and algorithms of the fractal dimensions used in the work, and an interpretation of local fractal dimensions of selected fragments of Figure 1.

**A1. Explanation of Figure. 1**

As has been explained in a previous study by Banaszak et al (2015), Figure 1 depicts six towns in a hexagon CPT pattern for space friction $\mu=0.09$. In this simulation experiment, we used a 2-dimentional grid. Because the x and y coordinates are set between -6 and 6, therefore we have a 12 by 12 field.

The starting points of the agent are located at equal intervals of $1/200$. We add one extra point to each direction where one of the coordinates is 0. As a consequence, there are 2,401 points on the x axis and also 2,401 points on the y axis, totaling in more than 5.76 million starting points for each value of the friction coefficient $\mu$. To obtain the graph of the fractal dimension as a function of $\mu$, we choose around 50 values of $\mu$. This means 300 million agent starting positions.

The agent starts from the bottom left corner (SW) and is attracted by six cities. Its trajectory is chaotic and the initial velocity decreases with respect to space friction and, consequently, energy dissipation. After many chaotic movements, the agent is attracted by one of the six cities, then the square from which it started is marked with the color of the city that attracted it. Further, the agent starts to move from the adjacent square in the same row. The procedure ends when the agent reaches the square in the top right corner (NE).

Figure 1 is a picture of order and spatial chaos. Order represents the inside of the hexagon divided into six equal sectors. Chaos is specified by a system of alternating, different-colored layers with extremely complex shapes. Between two layers (whose boundaries are fractals), there is always a layer of a different color. This sequence of distinctive layers occurs at each close-up (zoom) level and tends to infinity.

**A2. The theoretical Framework of Box-counting and Ruler Dimensions**

The fractal dimension, introduced as a systemic concept for learning by Mandelbrot (1977), has become a popular concept and has generated a large number of scientific publications. It has also found application in spatial and urban dynamics (see e.g., Nijkamp and Reggiani, 1992; Batty, 2005; Zanette, 2008; Zatos, 2015).

As has been noted above, in this study, the Box dimension ($d_{b}$) is used to analyze the complexity of gravitational fractals, and the Ruler dimension ($d_{r}$) is used for comparative purposes. The description of the theoretical foundations of these dimensions has been limited in this work to the necessary minimum. To this end, the authors fully share the view expressed in the work by Falconer (1997) “... it is more important to communicate ideas and concepts than technical detail. Too often in mathematical writing simple but elegant ideas are concealed by excessive generality”.

The description of the fractal dimension is based on the metric and Hausdorff dimensions (Li, Arneodo & Nekka, 2004). Let 𝐹 be any subset of the plane $\mathbb{R}^{2}$ and {$U_{i}$} its $\delta$-coverage, i.e., sets $U_{i}\epsilon\mathbb{R}^{2}$ covering $F$ have a diameter $|U_{i}|$ not bigger than $\delta$. We can now define a number:

$$\mathcal{H}_{\delta}^{s}\left( F \right)=\inf\left\{ \sum_{i} \left| U_{i} \right|^{s} \right\},$$

Then 𝑠-dimensional Hausdorff measure is:

$$\mathcal{H}^{s}(F)=\lim_{\delta\overset{\to}{}0} \mathcal{H}_{\delta}^{s}\left( F \right).$$

The Hausdorff measure is a generalization of familiar concepts: length, area or volume. For any Borel subset 𝐹 (i.e., a countable sum of open sets) of spaces $\mathbb{R}^{2}$ measure $\mathcal{H}_{s}(F)$ is directly proportional to the 2-dimensional volume ${vol}_{2}(F)$ of the set $F$ (area of the set *F*).

The Hausdorff dimension is defined as the critical value of 𝑠 for the Hausdorff measure, namely:

$$\dim_{H} F=\inf\left\{ s\geq0: \mathcal{H}^{s}\left( F \right)=0 \right\}=\sup\left\{ s: \mathcal{H}^{s}\left( F \right)=\infty\right\}.$$

In practice, the use of the above definition is very difficult. Therefore, in this paper, the authors apply (in a limit equivalent to Hausdorff dimension) the definition of the Box and Ruler dimensions.

Let $N_{\delta}(F)$ denote the smallest number of sets of $\delta$-coverage of the set concerned. The Box dimension is:

$$d_{b}=\lim_{\delta\overset{\to}{}0} \frac{\log N_{\delta}\left( F \right)}{-\log\delta}.$$

For the boundary of subset $F$ of plane ℝ^2^, its length $D(r)$ can be determined as a multiple of 𝑁(𝑟) of a certain fixed length 𝑟 (ruler), according to the following formula:

$$D\left( r \right)\propto Cr^{1-d}=N\left( r \right)r,$$

where (at the border crossing) $d$ is called the ruler dimension, and 𝐶 is the proportionality constant. Hence, we can assume that the ruler dimension is expressed by the formula:

$$d_{r}=\lim_{r\overset{\to}{}0} \frac{\log N(r)}{-\log r}.$$

In this study, we will interpret the coverage of the $F$ set, i.e., the area of the hexagonal influence of six cities, as a grid of squares corresponding to the size of one pixel of the image of the area obtained in our simulations. Consequently, the size $r$ of the Ruler is equal to the side length of the square of the grid at hand.

**A3. Identification of the Fractal Dimension of Gravity Fractals**

The gravitational fractal shown in Figure 1 is clearly the result of the use of a deterministic equation of motion (6). According to our equation, a characteristic spatial structure can be created that captures the complexity described by fractal dimensions. These methods of determining fractal dimensions of any area of an image composed of 𝑛 × 𝑛 pixels are presented on a randomly selected fragment of a fractal (see for illustrations, Figure 2). The value of the fractal dimension below the value of 1.0 means that the dimension was not calculated within the limit but with a fixed resolution, i.e., the adopted size of the box calculation.

**A3.1. Ruler Dimension**

The Ruler dimension is used here to establish the boundary of the hexagon attraction basins of the urban conurbations in the hexagonal system. The fractal which forms the edge of the attraction basins of the six cities is a line that separates differently colored pixels. For any pixel (𝑖, 𝑗), we check the number of the adjacent pixels of a different color, where we only include those along the sides^^[[1]](#footnote-1)^^. Let 𝑁 be the set of natural numbers $\{1,2,\ldots, n\}$. This results in a function $p: N^{2} \longrightarrow\{0,1,2,3,4\}$ assigning a number to the pixel’s sides adjacent to pixels of a different color, whose values will be denoted by $p_{i, j}$. For any image with dimensions $n \times n$ we calculate the expression:

$$P_{r}=\sum_{i,j=1}^{n} p_{i,j}.$$

In addition to pixels from the square side of the image, we need to assume the maximum value of 𝑝_𝑖,𝑗_  equal to 3 and for pixels from the corners of the image – 2. Because each fragment of the tested fractal consists of segments that are also the shared sides of two adjacent pixels, the number $P_{r}/2$ is the length of the entire boundary contained within the pixel matrix of dimension $N$. Thus, with the accuracy of the image size, we can assume that the ruler dimension of the boundary is given by the following equation:

$$D_{r}=\frac{\log\left( {P_{r}}/2 \right)}{\log\left( n \right)}.$$

We will now discuss Figure A1 for illustrative purposes.

**A3.2. Box Dimension**

We will now also calculate the box dimension of the fractal line, separating the attraction basins by creating a square grid of pixels, shifted from the image pixels by the vector $\left[ \frac{1}{2},\frac{1}{2} \right]$. Then, the center of each pixel is the common vertex of the four pixels of the image in question. The related illustration is provided in Figure A2. We determine the number of the grid pixels that cover the fractal. We only have information about the pixel color in the picture. For each of the four pixels: $\{(i-1, j-1), (i-1, j), (i, j-1), (i, j)\}$, we check if the line under consideration passes through their shared vertex fractal. Next, we create a function that assigns each pixel $(i, j)$ to a value of $p_{i,j}$ equal to 0 when the following pairs $\{(i-1, j-1), (i-1, j)\}$, $\{(i-1, j-1), (i, j-1)\}$, $\{(i-1, j-1), (i, j)\}$ are composed of pixels with the same colors, whereas it has a value of 1 when at least one of these pairs are pixels of different colors. So, the sum

$$P_{b}=\sum_{i,j=2}^{n} p_{i,j},$$

counts all the pixels (square grid) covering a fractal. Inside a picture with dimensions $n\times n$, the created mesh has the dimensions $(n-1)\times(n-1)$. The dimension Box, with the accuracy of the image size, can then be determined using the formula:

$$D_{b}=\frac{\log\left( P_{b} \right)}{\log\left( n-1 \right)}.$$

Note that inside each pixel of the created grid, the length of the fractal line can take the values: $0, 1, 1.5, 2$. So, the box dimension will be less than the ruler dimension and never exceed $\frac{\log\left( n-1 \right)^{2}}{\log\left( n-1 \right)}=2.$

The box dimension of the attraction basin of a specific city treated as an irregular figure is illustrated in Figure A3.

The method of determining the box size of an irregular figure, which is a basin of attraction for each city, is much simpler. Calculate the number of all pixels of the given color $k$:

$$P_{a}\left( k \right)=\sum_{i,j=1}^{n} p_{i,j}\left( k \right),$$

where $p_{i, j}\left( k \right)=1$ if the pixel is the color 𝑘 and $p_{i, j}\left( k \right)=0$ otherwise. Then, the Box dimension of this area with the accuracy of the image size can be represented by the following formula:

$$D_{a}=\frac{\log\left( P_{a}\left( k \right) \right)}{\log\left( n \right)}.$$

In this way, we can describe the complexity of gravitational fractals in terms of global and local dimensions. They are presented in Figure A3.

**A4. Local fractal dimensions in fragments B, C and E**

Figure 1 also shows fragments A, B, C, D and E that are characteristic in terms of structure and shape. Two of them, A and D, are analyzed in the main part of this study whereas the structural properties of fragments B, C and E are discussed below.

**Figure A1. Presentation of the method of determining the ruler dimension of the boundary of the snapping areas (from the left, the pixel lines: at the vertex, at the side and in the center of the grid).**

**A**

**B**

**C**

**D**

**E**

Legend: **(A)** Randomly selected fragment with a bold line representing the boundary of the attraction basins**. (B)** Boundary of the attraction basins with selected pixels: vertex, edge, internal. **(C-E)** Magnification of each of the major pixel types (vertex, edge, inner) adjacent to a boundary line.

**Figure A2. The geometric presentation of the method for determining the box dimension of areas of attraction.**

***(i, j)***

***(i, j-1)***

***(i-1, j)***

***(i-1, j-1)***

**A**

**B**

**C**

Legend: **(A)** Pixel and subpixel grid in randomly selected fragment of fractal.**(B)** Lines representing the boundaries of attraction basins of each cities in a selected fragment.**(C)** Subpixel covering 4 adjacent pixels .

**Figure A3. The geometric image of the method of determining the box dimension of the attraction basins as a geometric irregular figure.**

**A**

**B**

Legend: **(A)** Randomly selected fragment of fractal. (B) Attraction basins of city 3 (green color) lying within selected fragment

|  |  |  |  |  |  |  |
| --- | --- | --- | --- | --- | --- | --- |
|  |  |  |  |  |  |  |
|  |  |  |  |  |  |  |
|  |  |  |  |  |  |  |
|  |  |  |  |  |  |  |
|  |  |  |  |  |  |  |
|  |  |  |  |  |  |  |
|  |  |  |  |  |  |  |
|  |  |  |  |  |  |  |
|  |  |  |  |  |  |  |
|  |  |  |  |  |  |  |
|  |  |  |  |  |  |  |
|  |  |  |  |  |  |  |
|  |  |  |  |  |  |  |
|  |  |  |  |  |  |  |
|  |  |  |  |  |  |  |
|  |  |  |  |  |  |  |
|  |  |  |  |  |  |  |
|  |  |  |  |  |  |  |
|  |  |  |  |  |  |  |
|  |  |  |  |  |  |  |
| 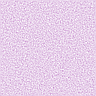 |  |  |  |  |  |  |
|  |  |  |  |  |  |  |
| 1 | 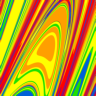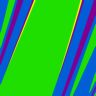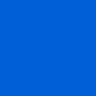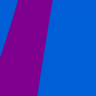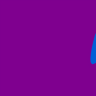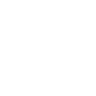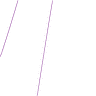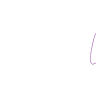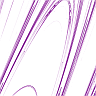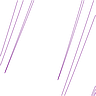 |  |  |  |  |  |
|  |  |  |  |  |  |  |
|  |  |  |  |  |  |  |
|  |  |  |  |  |  |  |
|  | 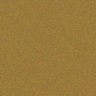 |  |  |  |  |  |
| 7 |  |  |  |  |  |  |
|  |  |  |  |  |  |  |
|  |  |  |  |  |  |  |
| μ | 0.02 | 0.09 | 0.16 | 0.26 | 0.36 | 0.46 |

**Figure A4. Variability of the local box dimension of the boundary of the attraction basins of each city in fragment B depending on coefficient** $\boldsymbol{\mu}$**.**

It is easy to state that with increasing space resistance, that is, the values of coefficient $\mu$, the boundaries of the attraction basins of the individual cities become increasingly smoothed, and the values of their fractal dimension tend towards unity. We note that between $\mu=0.16$ and $\mu=0.21$, the fractal dimension of the boundaries of the attraction basins of all cities successively reaches the value of zero. This means that the attraction basins of the individual cities disappear one by one. However, the entire fragment B may be marked with one color. With further increase in the resistance of space, only two cities begin to dominate the space of fragment B. To a greater extent, city 1 (purple) and to a smaller extent city 2 (blue).

|  |  |  |  |  |  |  |
| --- | --- | --- | --- | --- | --- | --- |
|  |  |  |  |  |  |  |
|  |  |  |  |  |  |  |
|  |  |  |  |  |  |  |
|  |  |  |  |  |  |  |
|  |  |  |  |  |  |  |
|  |  |  |  |  |  |  |
|  |  |  |  |  |  |  |
|  |  |  |  |  |  |  |
|  |  |  |  |  |  |  |
|  |  |  |  |  |  |  |
|  |  |  |  |  |  |  |
|  |  |  |  |  |  |  |
|  |  |  |  |  |  |  |
|  |  |  |  |  |  |  |
|  |  |  |  |  |  |  |
|  |  |  |  |  |  |  |
|  |  |  |  |  |  |  |
|  |  |  |  |  |  |  |
|  | 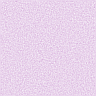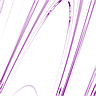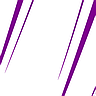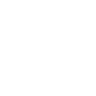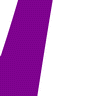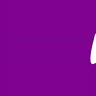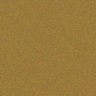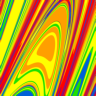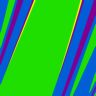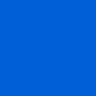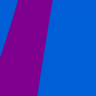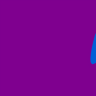 |  |  |  |  |  |
|  |  |  |  |  |  |  |
| 1 |  |  |  |  |  |  |
|  |  |  |  |  |  |  |
|  |  |  |  |  |  |  |
|  |  |  |  |  |  |  |
|  |  |  |  |  |  |  |
| 7 |  |  |  |  |  |  |
|  |  |  |  |  |  |  |
|  |  |  |  |  |  |  |
| μ | 0.02 | 0.09 | 0.16 | 0.26 | 0.36 | 0.46 |

**Figure A5. Local fractal dimension of the attraction basin of each city as an entire non-regular figure in fragment B.**

When the resistance of space is extremely small, e.g., $\mu=0.005$, all attraction basins have the same fractal dimension, i.e., $d_{b}=1.769$. With an increase of space resistance, fractal dimensions of the three cities, – i.e. 1. (purple), 2. (blue) and 3. (green) – first noticeably decrease, then they grow and then decrease again to value $d_{b}=0$.An exception is city 2. (blue) which is the only dominating one among the six cities, i.e., dominant over the space of fragment B in the range $0.22\leq\mu\leq0.3$. The fractal dimension of its attraction basin is then 2. Beginning with the value of $\mu\geq0.32$ the competition of city 1 is revealed (purple). At the value of $\mu=0.38$ it begins to dominate the space of fragment B, which it finally captures.

|  |  |  |  |  |  |  |
| --- | --- | --- | --- | --- | --- | --- |
|  |  |  |  |  |  |  |
|  |  |  |  |  |  |  |
|  |  |  |  |  |  |  |
|  |  |  |  |  |  |  |
|  |  |  |  |  |  |  |
|  |  |  |  |  |  |  |
|  |  |  |  |  |  |  |
|  |  |  |  |  |  |  |
|  |  |  |  |  |  |  |
|  |  |  |  |  |  |  |
|  |  |  |  |  |  |  |
|  |  |  |  |  |  |  |
|  |  |  |  |  |  |  |
|  |  |  |  |  |  |  |
|  |  |  |  |  |  |  |
|  |  |  |  |  |  |  |
|  |  |  |  |  |  |  |
|  |  |  |  |  |  |  |
| 6 |  |  |  |  |  |  |
|  |  |  | 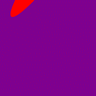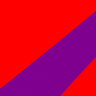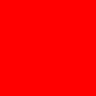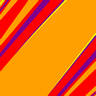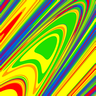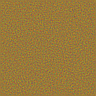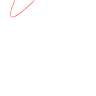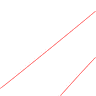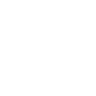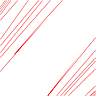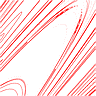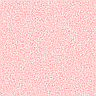 |  |  |  |
|  |  |  |  |  |  |  |
|  |  |  |  |  |  |  |
|  |  |  |  |  |  |  |
| 7 |  |  |  |  |  |  |
|  |  |  |  |  |  |  |
|  |  |  |  |  |  |  |
| μ | 0.02 | 0.09 | 0.16 | 0.26 | 0.36 | 0.46 |

**Figure A6. Variability of the local box dimension of the boundary of the attraction basins of each city in fragment C depending on coefficient μ.**

Figure A6 shows the distribution of fractal dimensions of the attraction basins of the individual cities. In the case of a very small space resistance ($\mu=0.02$) – when the interactions of cities on the agents are fully chaotic – the fractal dimension of the boundaries of the attraction basins is very high and amounts to $d_{b}=1.915$. As the resistance of space increases, i.e., following an increase in transport costs, chaos gradually gives way to order and the values of $d_{b}$ drop to zero, as individual attraction basins disappear, except for the red one (city 6), which fills the entire surface of fragment C. This indicates full domination of one city in space C. However, from the value $\mu=0.32$, onwards city 1. (purple) again begins to compete for the space of fragment C and ultimately dominates the majority of it. This process is illustrated by the icons in Fig. A6.

|  |  |  |  |  |  |  |
| --- | --- | --- | --- | --- | --- | --- |
|  |  |  |  |  |  |  |
|  |  |  |  |  |  |  |
|  |  |  |  |  |  |  |
|  |  |  |  |  |  |  |
|  |  |  |  |  |  |  |
|  |  |  |  |  |  |  |
|  |  |  |  |  |  |  |
|  |  |  |  |  |  |  |
|  |  |  |  |  |  |  |
|  |  |  |  |  |  |  |
|  |  |  |  |  |  |  |
|  |  |  |  |  |  |  |
|  |  |  |  |  |  |  |
|  |  |  |  |  |  |  |
|  |  |  |  |  |  |  |
|  |  |  |  |  |  |  |
|  |  |  |  |  |  |  |
|  |  |  |  |  |  |  |
| 6 | 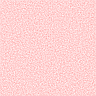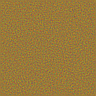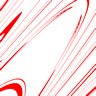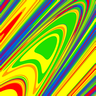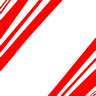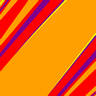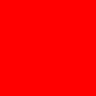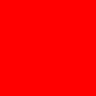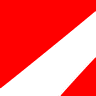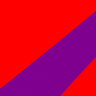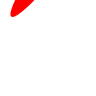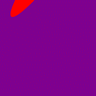 |  |  |  |  |  |
|  |  |  |  |  |  |  |
|  |  |  |  |  |  |  |
|  |  |  |  |  |  |  |
|  |  |  |  |  |  |  |
| 7 |  |  |  |  |  |  |
|  |  |  |  |  |  |  |
| μ | 0.02 | 0.09 | 0.16 | 0.26 | 0.36 | 0.46 |

**Figure A7. Local fractal dimensions of the attraction basin of each city as an entire non-regular figure in fragment C.**

In a situation where the fractal boundaries of the attraction basins lose their fractal properties (by approaching a straight line), the intertwined layers of colors in the examined fragment can be treated as separate, complex geometric figures with a certain fractal dimension. In extreme cases, the tested fragment can be filled with only one color and then $d_{b}=2$. This is the case in Figure A7. When $\mu=0.005$ – all cities have an equal share in the influence on the agent and then $d_{b}=1.77$. However, from $\mu=0.16$, some cities fall out of the competition for the space of fragment C. In the range of $0.22\leq\mu\leq0.3$, in space C, city 6 (red) dominates. However, with a further increase in space resistance, this city loses its dominance to city 1 (purple), which ultimately gains dominance among all the cities affecting the agent in space C.

| 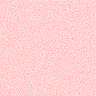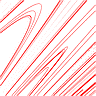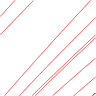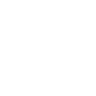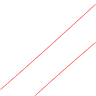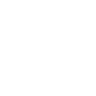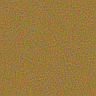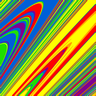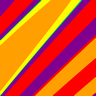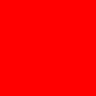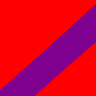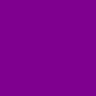 |  |  |  |  |  |  |
| --- | --- | --- | --- | --- | --- | --- |
|  |  |  |  |  |  |  |
|  |  |  |  |  |  |  |
|  |  |  |  |  |  |  |
|  |  |  |  |  |  |  |
|  |  |  |  |  |  |  |
|  |  |  |  |  |  |  |
|  |  |  |  |  |  |  |
|  |  |  |  |  |  |  |
|  |  |  |  |  |  |  |
|  |  |  |  |  |  |  |
|  |  |  |  |  |  |  |
|  |  |  |  |  |  |  |
|  |  |  |  |  |  |  |
|  |  |  |  |  |  |  |
|  |  |  |  |  |  |  |
|  |  |  |  |  |  |  |
|  |  |  |  |  |  |  |
|  |  |  |  |  |  |  |
|  |  |  |  |  |  |  |
|  |  |  |  |  |  |  |
| 6 |  |  |  |  |  |  |
|  |  |  |  |  |  |  |
|  |  |  |  |  |  |  |
|  |  |  |  |  |  |  |
|  |  |  |  |  |  |  |
| 7 |  |  |  |  |  |  |
|  |  |  |  |  |  |  |
| μ | 0.02 | 0.09 | 0.16 | 0.26 | 0.36 | 0.46 |

**Figure A8. Variability of the local box dimension of the boundary of the attraction basins of each city in fragment E, depending on coefficient μ.**

This figure presents the history of the influence on the agent of individual cities in fragment E. Basically, it is similar to the ones occurring in fragments B and C in the sense that there is a gradual transition from chaotic to orderly interactions with an increasing value of $\mu$. In the range $0.22\leq\mu\leq0.34$ , city 6 (red) and city 1 (purple) with the value $\mu=0.38$ become dominant in space E. Therefore, also in fragment E, city 1 dominates over its entire space in the final phase of the spatial interaction process. This should be understood in such a way that for the value $\mu=0.52$, the attraction basins of the cities in the area under consideration are already established and do not change. However, beyond the area of Figure 1, the process of spatial interactions may still occur. This is an obvious consequence of the fact that fragments B, C and E are in the city 1 (purple) attraction basin (see Figure 1). With respect to the symmetry of the hexagon, a similar variation in fractal dimensions will occur in the area of the remaining attraction basins.

|  |  |  |  |  |  |  |
| --- | --- | --- | --- | --- | --- | --- |
|  |  |  |  |  |  |  |
|  |  |  |  |  |  |  |
|  |  |  |  |  |  |  |
|  |  |  |  |  |  |  |
|  |  |  |  |  |  |  |
|  |  |  |  |  |  |  |
|  |  |  |  |  |  |  |
|  |  |  |  |  |  |  |
|  |  |  |  |  |  |  |
|  |  |  |  |  |  |  |
|  |  |  |  |  |  |  |
|  |  |  |  |  |  |  |
|  |  |  |  |  |  |  |
|  |  |  |  |  |  |  |
|  |  |  |  |  |  |  |
|  |  |  |  | 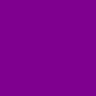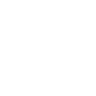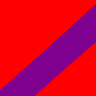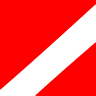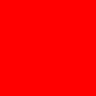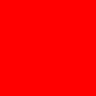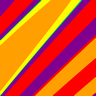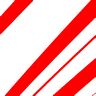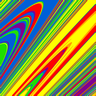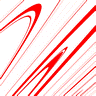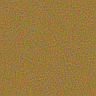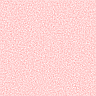 |  |  |
| 6 |  |  |  |  |  |  |
|  |  |  |  |  |  |  |
|  |  |  |  |  |  |  |
|  |  |  |  |  |  |  |
|  |  |  |  |  |  |  |
| 7 |  |  |  |  |  |  |
|  |  |  |  |  |  |  |
|  |  |  |  |  |  |  |
| μ | 0.02 | 0.09 | 0.16 | 0.26 | 0.36 | 0.46 |

**Figure A9. Local fractal dimensions of the attraction basin of each city as an entire non-regular figure in fragment E.**

As has been specified above, the spatial structure of the attraction basins of the individual cities changes with the constraints associated with the resistance of space. Figure A9 illustrates the variability in fragment E. What attracts our attention is a large variation in the complexity of the attraction basins of the individual cities in the range $0.04\leq\mu\leq0.22$. The value $\mu=0.22$ is critical for city 1 (purple), 2 (blue), 3 (green), 4 (yellow) and 5 (orange) because they lose their influence on the space of fragment E. City 6 (red) becomes fully dominant in this fragment. However, beginning with the value $\mu=0.38$ in space E, city 1 (purple) starts to dominate and in the final phase of the spatial interactions process – the dimension of its attraction basin is 2, and for the remaining cities 0.

|  |  |  |  |  |  |  |
| --- | --- | --- | --- | --- | --- | --- |
|  |  |  |  |  |  |  |
|  |  |  |  |  |  |  |
|  |  |  |  |  |  |  |
|  |  |  |  |  |  |  |
|  |  |  |  |  |  |  |
|  |  |  |  |  |  |  |
|  |  |  |  |  |  |  |
|  |  |  |  |  |  |  |
|  |  |  |  |  |  |  |
|  |  |  |  |  |  |  |
|  |  |  |  |  |  |  |
|  |  |  |  |  |  |  |
|  |  |  |  |  |  |  |
|  |  |  |  |  |  |  |
|  | 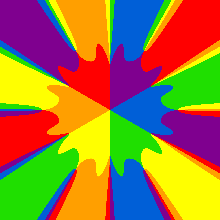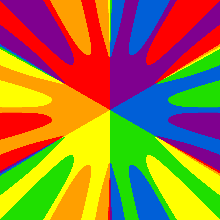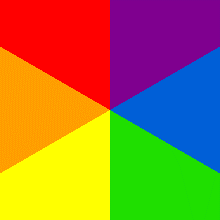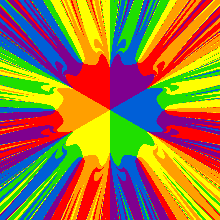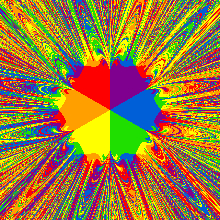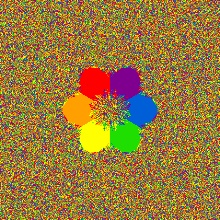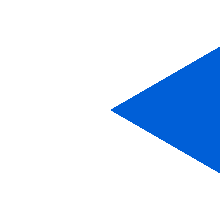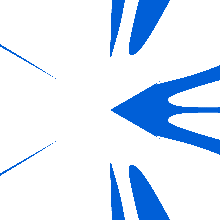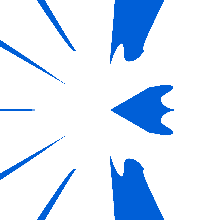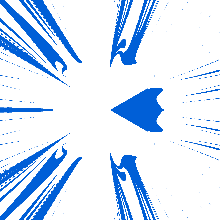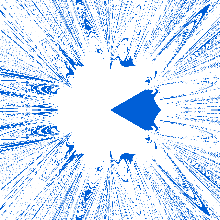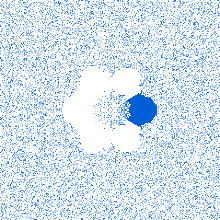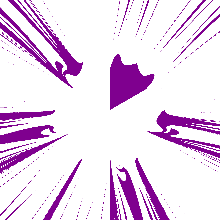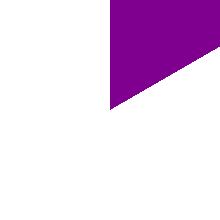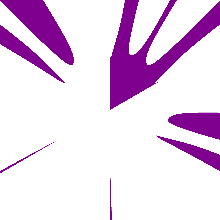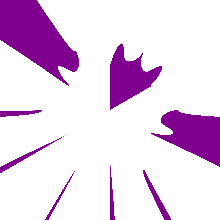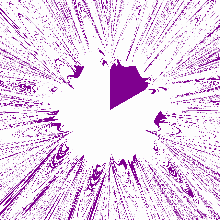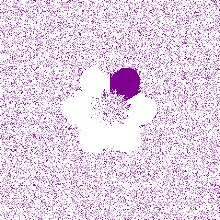 |  |  |  |  |  |
|  |  |  |  |  |  |  |
| 1 |  |  |  |  |  |  |
|  |  |  |  |  |  |  |
|  |  |  |  |  |  |  |
|  |  |  |  |  |  |  |
|  |  |  |  |  |  |  |
| 2 |  |  |  |  |  |  |
|  |  |  |  |  |  |  |
|  |  |  |  |  |  |  |
|  |  |  |  |  |  |  |
|  |  |  |  |  |  |  |
| 7 |  |  |  |  |  |  |
|  |  |  |  |  |  |  |
|  |  |  |  |  |  |  |
| μ | 0.005 | 0.09 | 0.17 | 0.28 | 0.38 | 0.52 |

**Figure A10. Global dimension of the attraction basin of each city as an entire non-regular figure.**

Figure A10 shows the variability of the box dimension ($d_{b}$) and the attraction basins of cities 1, …, 6 over the entire area of Figure 1 – depending on the value of $\mu$. The mean value of this dimension is represented by the black line ($\overline{d}_{b}=1.77$). This is a high value and demonstrates high structural complexity of the attraction basins. Figure A10 also confirms the fact that when spatial interactions in the hexagon (Figure 1) are modeled on the square’s surface, then the final attraction basins of cities 1, 3, 4 and 6 have larger areas than cities 2 and 5. Hence, the fractal dimension lines split twice with values of $\mu=0.08$ and 0.185.

**Table A1. Box fractal dimensions of boundaries of attraction basins in fragment B.**

| Box dimension $d_{b}$ | | | | | | |
| --- | --- | --- | --- | --- | --- | --- |
| μ | 1-purple | 2-blue | 3-green | 4-yellow | 5-orange | 6-red |
| 0.005 | 1.9151 | 1.9153 | 1.9153 | 1.9153 | 1.9154 | 1.9153 |
| 0.010 | 1.9153 | 1.9153 | 1.9152 | 1.9152 | 1.9152 | 1.9154 |
| 0.015 | 1.9153 | 1.9153 | 1.9152 | 1.9154 | 1.9152 | 1.9152 |
| 0.020 | 1.9153 | 1.9153 | 1.9151 | 1.9151 | 1.9152 | 1.9152 |
| 0.025 | 1.9150 | 1.9154 | 1.9154 | 1.9153 | 1.9147 | 1.9149 |
| 0.030 | 1.9139 | 1.9129 | 1.9140 | 1.9155 | 1.9157 | 1.9146 |
| 0.035 | 1.9127 | 1.9103 | 1.9104 | 1.9121 | 1.9144 | 1.9148 |
| 0.040 | 1.9104 | 1.9108 | 1.9098 | 1.9049 | 1.9042 | 1.9049 |
| 0.045 | 1.9022 | 1.9143 | 1.9114 | 1.8989 | 1.8904 | 1.8909 |
| 0.050 | 1.8933 | 1.9021 | 1.8971 | 1.8903 | 1.8814 | 1.8846 |
| 0.055 | 1.8834 | 1.8802 | 1.8737 | 1.8695 | 1.8652 | 1.8757 |
| 0.060 | 1.8634 | 1.8469 | 1.8401 | 1.8472 | 1.8501 | 1.8628 |
| 0.065 | 1.8305 | 1.8075 | 1.8019 | 1.8206 | 1.8378 | 1.8433 |
| 0.070 | 1.7894 | 1.7593 | 1.7609 | 1.7964 | 1.8228 | 1.8189 |
| 0.075 | 1.7365 | 1.7012 | 1.7147 | 1.7656 | 1.8013 | 1.7882 |
| 0.080 | 1.6844 | 1.6319 | 1.6653 | 1.7307 | 1.7660 | 1.7468 |
| 0.085 | 1.6414 | 1.5840 | 1.6390 | 1.7006 | 1.7172 | 1.6968 |
| 0.090 | 1.6198 | 1.5730 | 1.6217 | 1.6680 | 1.6752 | 1.6621 |
| 0.095 | 1.5908 | 1.5502 | 1.6071 | 1.6441 | 1.6356 | 1.6248 |
| 0.100 | 1.5723 | 1.5295 | 1.5714 | 1.6071 | 1.5884 | 1.5848 |
| 0.105 | 1.5339 | 1.5088 | 1.5188 | 1.5419 | 1.5341 | 1.5345 |
| 0.110 | 1.4745 | 1.4660 | 1.4630 | 1.4640 | 1.4761 | 1.4833 |
| 0.115 | 1.4089 | 1.4287 | 1.4556 | 1.4215 | 1.4269 | 1.4445 |
| 0.120 | 1.3675 | 1.4192 | 1.4499 | 1.3892 | 1.3848 | 1.4062 |
| 0.125 | 1.3456 | 1.3987 | 1.4114 | 1.3350 | 1.3284 | 1.3588 |
| 0.130 | 1.3067 | 1.3565 | 1.3370 | 1.2683 | 1.2643 | 1.2719 |
| 0.135 | 1.3016 | 1.3036 | 1.2403 | 1.2325 | 1.2261 | 1.2310 |
| 0.140 | 1.2988 | 1.3017 | 1.2256 | 1.2203 | 1.2164 | 1.2164 |
| 0.145 | 1.2968 | 1.3028 | 1.2289 | 1.2180 | 1.2052 | 1.2056 |
| 0.150 | 1.2948 | 1.3139 | 1.2501 | 1.2159 | 1.1750 | 1.1760 |
| 0.155 | 1.2928 | 1.3258 | 1.2661 | 1.2082 | 1.1236 | 1.1246 |
| 0.160 | 1.2804 | 1.3306 | 1.2717 | 1.1786 | 0.9895 | 0.9918 |
| 0.170 | 1.2247 | 1.2993 | 1.2191 | 0.9977 | 0 | 0 |
| 0.180 | 1.0739 | 1.2029 | 1.1443 | 0 | 0 | 0 |
| 0.190 | 0 | 1.1569 | 1.1569 | 0 | 0 | 0 |
| 0.200 | 0 | 1.1362 | 1.1362 | 0 | 0 | 0 |
| 0.210 | 0 | 1.0302 | 1.0302 | 0 | 0 | 0 |
| 0.220 | 0 | 0 | 0 | 0 | 0 | 0 |
| 0.230 | 0 | 0 | 0 | 0 | 0 | 0 |
| 0.240 | 0 | 0 | 0 | 0 | 0 | 0 |
| 0.250 | 0 | 0 | 0 | 0 | 0 | 0 |
| 0.260 | 0 | 0 | 0 | 0 | 0 | 0 |
| 0.270 | 0 | 0 | 0 | 0 | 0 | 0 |
| 0.280 | 0 | 0 | 0 | 0 | 0 | 0 |
| 0.290 | 0 | 0 | 0 | 0 | 0 | 0 |
| 0.300 | 0 | 0 | 0 | 0 | 0 | 0 |
| 0.320 | 0.9403 | 0.9403 | 0 | 0 | 0 | 0 |
| 0.340 | 1.0515 | 1.0515 | 0 | 0 | 0 | 0 |
| 0.360 | 1.0848 | 1.0848 | 0 | 0 | 0 | 0 |
| 0.380 | 1.0604 | 1.0604 | 0 | 0 | 0 | 0 |
| 0.400 | 1.0285 | 1.0285 | 0 | 0 | 0 | 0 |
| 0.420 | 1.0306 | 1.0306 | 0 | 0 | 0 | 0 |
| 0.440 | 1.0326 | 1.0326 | 0 | 0 | 0 | 0 |
| 0.460 | 0.8961 | 0.8961 | 0 | 0 | 0 | 0 |
| 0.480 | 0 | 0 | 0 | 0 | 0 | 0 |

This table contains the values of the local fractal dimension of the attraction basins, separately for individual cities, depending on the values of $\mu$. When space resistance is low – no city is privileged and each city attracts an agent. Hence, the local fractal dimension of the boundaries of the attraction basins of these cities is in fact identical and amounts to $d_{b}=1.915$.

**Table A2. Box fractal dimension of attraction basins treated as an entire irregular figure in fragment B.**

| Box dimension $d_{b}$ | | | | | | |
| --- | --- | --- | --- | --- | --- | --- |
| μ | violet | blue | green | yellow | orange | red |
| 0.005 | 1.7696 | 1.7698 | 1.7699 | 1.7699 | 1.7699 | 1.7697 |
| 0.010 | 1.7699 | 1.7698 | 1.7698 | 1.7697 | 1.7698 | 1.7699 |
| 0.015 | 1.7699 | 1.7698 | 1.7697 | 1.7699 | 1.7698 | 1.7697 |
| 0.020 | 1.7699 | 1.7700 | 1.7697 | 1.7697 | 1.7697 | 1.7698 |
| 0.025 | 1.7697 | 1.7702 | 1.7702 | 1.7700 | 1.7693 | 1.7695 |
| 0.030 | 1.7691 | 1.7676 | 1.7692 | 1.7714 | 1.7716 | 1.7699 |
| 0.035 | 1.7696 | 1.7672 | 1.7681 | 1.7690 | 1.7719 | 1.7729 |
| 0.040 | 1.7762 | 1.7754 | 1.7744 | 1.7645 | 1.7636 | 1.7640 |
| 0.045 | 1.7703 | 1.7872 | 1.7856 | 1.7667 | 1.7510 | 1.7535 |
| 0.050 | 1.7745 | 1.7819 | 1.7754 | 1.7700 | 1.7480 | 1.7664 |
| 0.055 | 1.7844 | 1.7698 | 1.7611 | 1.7621 | 1.7532 | 1.7849 |
| 0.060 | 1.7935 | 1.7462 | 1.7423 | 1.7591 | 1.7830 | 1.7855 |
| 0.065 | 1.7782 | 1.7276 | 1.7165 | 1.7600 | 1.8200 | 1.7874 |
| 0.070 | 1.7366 | 1.6817 | 1.7123 | 1.7721 | 1.8427 | 1.8039 |
| 0.075 | 1.6844 | 1.6571 | 1.6936 | 1.7973 | 1.8610 | 1.7979 |
| 0.080 | 1.6379 | 1.6567 | 1.6924 | 1.8131 | 1.8703 | 1.7812 |
| 0.085 | 1.6052 | 1.6641 | 1.7337 | 1.8257 | 1.8606 | 1.7639 |
| 0.090 | 1.6279 | 1.6817 | 1.7427 | 1.8502 | 1.8283 | 1.7576 |
| 0.095 | 1.6514 | 1.6926 | 1.7716 | 1.8618 | 1.7794 | 1.7587 |
| 0.100 | 1.6873 | 1.6991 | 1.7965 | 1.8604 | 1.7320 | 1.7558 |
| 0.105 | 1.7214 | 1.7075 | 1.8106 | 1.8708 | 1.6521 | 1.7292 |
| 0.110 | 1.7531 | 1.7123 | 1.8337 | 1.8642 | 1.5856 | 1.6844 |
| 0.115 | 1.7643 | 1.7224 | 1.8658 | 1.8376 | 1.5493 | 1.6402 |
| 0.120 | 1.7673 | 1.7227 | 1.9053 | 1.7815 | 1.5054 | 1.5822 |
| 0.125 | 1.7675 | 1.7116 | 1.9368 | 1.6754 | 1.4358 | 1.5351 |
| 0.130 | 1.7685 | 1.6913 | 1.9512 | 1.5773 | 1.3740 | 1.4634 |
| 0.135 | 1.7670 | 1.6998 | 1.9540 | 1.5468 | 1.3232 | 1.3552 |
| 0.140 | 1.7591 | 1.7372 | 1.9508 | 1.5364 | 1.2928 | 1.2991 |
| 0.145 | 1.7494 | 1.7655 | 1.9476 | 1.5277 | 1.2522 | 1.2584 |
| 0.150 | 1.7381 | 1.7924 | 1.9435 | 1.5164 | 1.1922 | 1.1989 |
| 0.155 | 1.7240 | 1.8167 | 1.9389 | 1.4960 | 1.0955 | 1.1027 |
| 0.160 | 1.7044 | 1.8367 | 1.9353 | 1.4591 | 0.8797 | 0.8870 |
| 0.170 | 1.6312 | 1.8724 | 1.9280 | 1.1928 | 0 | 0 |
| 0.180 | 1.4271 | 1.9029 | 1.9157 | 0 | 0 | 0 |
| 0.190 | 0 | 1.9241 | 1.8963 | 0 | 0 | 0 |
| 0.200 | 0 | 1.9536 | 1.8467 | 0 | 0 | 0 |
| 0.210 | 0 | 1.9905 | 1.6601 | 0 | 0 | 0 |
| 0.220 | 0 | 2.0000 | 0 | 0 | 0 | 0 |
| 0.230 | 0 | 2.0000 | 0 | 0 | 0 | 0 |
| 0.240 | 0 | 2.0000 | 0 | 0 | 0 | 0 |
| 0.250 | 0 | 2.0000 | 0 | 0 | 0 | 0 |
| 0.260 | 0 | 2.0000 | 0 | 0 | 0 | 0 |
| 0.270 | 0 | 2.0000 | 0 | 0 | 0 | 0 |
| 0.280 | 0 | 2.0000 | 0 | 0 | 0 | 0 |
| 0.290 | 0 | 2.0000 | 0 | 0 | 0 | 0 |
| 0.300 | 0 | 2.0000 | 0 | 0 | 0 | 0 |
| 0.320 | 1.5750 | 1.9952 | 0 | 0 | 0 | 0 |
| 0.340 | 1.7727 | 1.9760 | 0 | 0 | 0 | 0 |
| 0.360 | 1.8861 | 1.9318 | 0 | 0 | 0 | 0 |
| 0.380 | 1.9211 | 1.8999 | 0 | 0 | 0 | 0 |
| 0.400 | 1.9404 | 1.8727 | 0 | 0 | 0 | 0 |
| 0.420 | 1.9557 | 1.8418 | 0 | 0 | 0 | 0 |
| 0.440 | 1.9719 | 1.7908 | 0 | 0 | 0 | 0 |
| 0.460 | 1.9985 | 1.4237 | 0 | 0 | 0 | 0 |
| 0.480 | 2.0000 | 0 | 0 | 0 | 0 | 0 |

Table A2 contains values of these kinds of dimensions while their distribution as a function of $\mu$ is shown in Figure A5.

1. ^1^ This is the case of the rook in chess. [↑](#footnote-ref-1)
